# Atypical SARS and *Escherichia coli* Bacteremia

**DOI:** 10.3201/eid1002.030501

**Published:** 2004-02

**Authors:** Thuan Tong Tan, Ban Hock Tan, Asok Kurup, Lynette Lin Ean Oon, Derrick Heng, Su Yun Se Thoe, Xin Lai Bai, Kwai Peng Chan, Ai Ee Ling

**Affiliations:** * Singapore General Hospital Infectious Diseases, Singapore

**Keywords:** atypical severe acute respiratory syndrome, SARS, dispatch

## Abstract

We describe a patient with severe acute respiratory syndrome (SARS) whose clinical symptoms were masked by *Escherichia coli* bacteremia. SARS developed in a cluster of healthcare workers who had contact with this patient. SARS was diagnosed when a chest infiltrate developed and when the patient’s brother was hospitalized with acute respiratory failure. We highlight problems in atypical cases and offer infection control suggestions.

Severe acute respiratory syndrome (SARS) is a newly recognized condition. In early March 2003, the World Health Organization (WHO) issued case definitions for SARS (*1*). In most studies, the clinical syndrome includes fever in 100% of patients (*2*–*4*). Other common clinical features include chills and rigors (73%), myalgia (60%), and cough (>50%). Some patients initially thought to have SARS have been excluded when tests showed other causes (*5*). We report a patient whose coexisting conditions masked the diagnosis of SARS, leading to a cluster of suspect and probable cases.

## Case Report

A 59-year-old Chinese man was admitted on March 24, 2003, to the Singapore General Hospital. He had previously been hospitalized in Tan Tock Seng Hospital, the hospital in which the first SARS outbreak in Singapore occurred (*6*), from March 5 to March 20 for diabetic nephropathy.

The patient had multiple coexisting conditions including ischemic heart disease with atrial fibrillation, previous stroke with scar epilepsy, diabetes mellitus with nephropathy (creatinine 242 μmol/L), and peripheral vascular disease. He was not on steroids or traditional medications.

Clinical signs and symptoms were melena and dizziness. He was pale, temperature was 36.5°C, blood pressure was 126/70 mm Hg, and pulse rate was 110/min. Chest examination was normal, and the abdomen was soft. Rectal examination showed melena. The patient also had a sloughy right heel ulcer. Laboratory values are shown in the Table. Antral gastritis was diagnosed on gastroscopy. Colonoscopy and barium enema were unsuccessful because of excessive fecal residue.

**Table:** Laboratory results for SARS patient^a^

Characteristic	Date			
	March 24	March 25	March 28	March 30
**Hemoglobin (g/dL)**	5.6	7.5	9.4	10.9
Erythrocyte count ( x10^9^/L)	11.3	8.99	8.39	10.07
**Polymorphs (%)**	79.3	83.0	77.3	81.4
Lymphocytes (%)	11.4	8.1	14.3	13.8
Monocytes (%)	8.1	5.3	8.2	4.5
Eosinophils (%)	1.1	0.6	0.1	0.1
Basophils (%)	0.1	0.3	0.1	0.2
**Platelets (x10^9^/L)**	421	459	332	286

^a^SARS, severe acute respiratory syndrome.

The patient had a temperature spike (38.4°C) on March 26, and intravenous (IV) amoxicillin/clavulanic acid was started. His temperature spiked again (38.8°C) on March 28. The source of sepsis was thought to be the necrotic heel ulcer; wound débridement was performed on March 30.

The fever persisted from March 28 until April 2. Blood cultures drawn on March 28 isolated *E. coli* of intermediate sensitivity to amoxicillin/clavulanic acid. Further evaluation for the source of bacteremia included urinalysis, which indicated a leukocyte count of 4 and erythrocyte count of 165. Ultrasonography showed a 2.8-cm abscess at the midpole of the right kidney. Urine culture yielded mixed bacteria growth. The patient’s medication was changed to IV ceftriaxone (to which the organism was susceptible) on April 1. Tissue cultures from the necrotic heel yielded *Pseudomonas aeruginosa* sensitive only to imipenem. Although fever was lower after 1 day of ceftriaxone, the patient’s medication was switched to IV imipenem on April 2. He remained afebrile thereafter.

**Figure 1:**
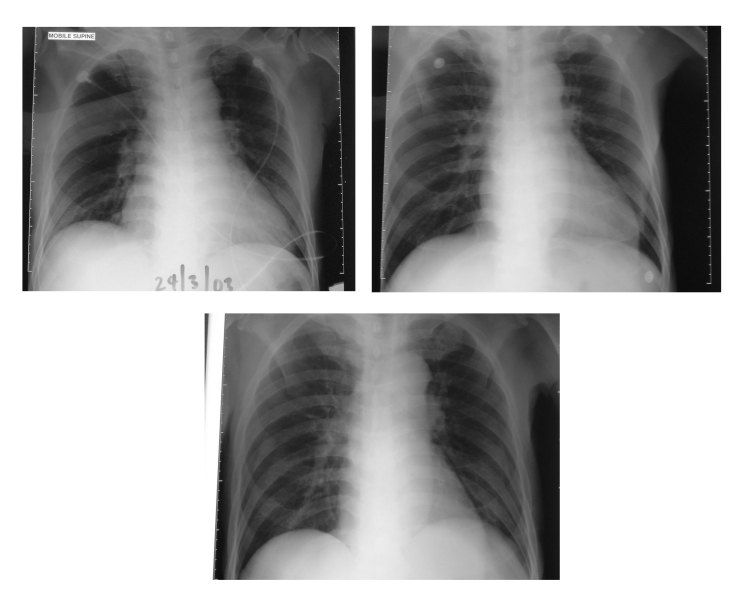
A, radiograph on admission; B, radiograph on day 5 of hospital stay; C, radiograph on day 7 of hospital stay.

On April 1 (6 days after the patient’s first spike of temperature), three healthcare workers from the ward into which he had first been admitted became febrile. At this time, physicians were notified that the patient was on a surveillance program for SARS. He was transferred from the general surgical ward to an isolation room, and healthcare workers used a combination of airborne, contact, and droplet precautions. His clinical course was scrutinized for evidence of SARS. Despite the positive contact history, he did not have any respiratory symptoms. Three chest x-rays performed on days 1, 5, and 7 of hospitalization were normal (Figure 1A, B, and C). The fever could have been attributed to the *E. coli* bacteremia because it subsided after the patient’s antimicrobial drug was changed to an appropriate one. Over the subsequent days, 16 healthcare workers from the two wards where this patient was treated became febrile.

**Figure 2:**
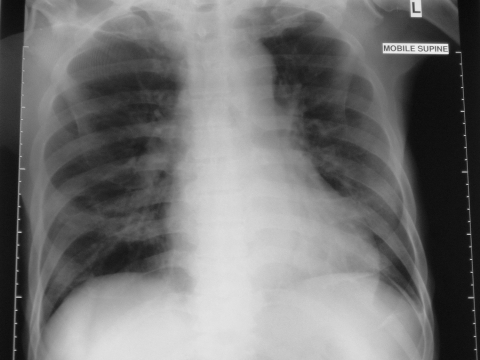
Radiograph on day 11 of hospital stay (day 14 after contact with a SARS patient).

On day 11 of hospitalization (April 3, 14 days after the patient’s last day in Tan Tock Seng Hospital), an ill-defined air space shadow was noted in the right lower zone of his chest x-ray (Figure 2). On May 5, he was transferred back to Tan Tock Seng Hospital, an officially designated SARS hospital. The patient had no further temperature spikes and no respiratory symptoms, despite the chest x-ray abnormalities. Respiratory distress did not develop, and neither methylprednisolone nor ribavirin was given. The patient completed a course of imipenem but remains in hospital at the time of writing because of nosocomial sepsis. On April 8, his brother was also admitted to Tan Tock Seng Hospital for acute respiratory failure and died.

Throat swabs from our patient were collected on April 4 and stool samples were colleted on April 10, days 9 and 15 after the onset of fever, respectively. These samples were sent for viral studies that included virus isolation and reverse transcriptase–polymerase chain reaction (RT-PCR) for the SARS-associated coronavirus (SARS-CoV). Three sets of primers were used. The first two sets were SAR1S/As and BNIoutS2/As as described in the paper by Drosten et al. (*7*); the third primer set was Cor1/2 (5′-CAC CGT TTC TAC AGG TTA GCT AAC GA-3′ and 5′-AAA TGT TTA CGC AGG TAA GCC TAA AA-3′) from the Government Virus Unit, Hong Kong.

From the throat swab, a weak band measuring 310 bp was found by using the Cor1/2 primer set only. Positive bands were seen with all three primer sets on the stool sample; the bands with the SAR1S/As and BNIoutS2/As primers measured 190 and 150 bp, respectively.

The diagnosis of SARS in the patient’s brother was subsequently confirmed on April 9 when a throat swab was positive for SARS-CoV by PCR. Multiple postmortem samples were also positive for SARS-CoV by PCR, and SARS-CoV was also isolated in the lung tissue.

Serum samples from the patient and the healthcare workers who were his contacts were tested for total antibodies to SARS-CoV with an enzyme immunoassay by using SARS-CoV Vero E6 cell lysate that had been developed by the Centers for Disease Control and Prevention. Results of serologic testing were positive at day 41. Serum samples were taken from 14 of the 16 healthcare workers at least 21 days after onset of symptoms. Of these, 13 were positive for antibodies to SARS-CoV.

Extensive epidemiologic studies identified this patient as the common source for the cluster of healthcare workers in Singapore General Hospital who were subsequently diagnosed with SARS. These healthcare workers were infected before chest infiltrates developed and the patient was isolated.

A total of 16 healthcare workers (13 nurses, one health attendant, one radiographer, and one doctor), 12 patients, and eight visitors (including his brother) from the wards in which the patient was admitted were eventually diagnosed with probable SARS. In addition, he was linked to a cluster of five healthcare workers (one radiographer and four health attendants), one visitor, and three outpatients at the diagnostic radiology department where he had barium studies and an ultrasound performed.

Epidemiologic evidence suggested that this patient was the source for this cluster. He was linked to one of the index cases in Tan Tock Seng Hospital, had the earliest onset of fever among the cohort of Singapore General Hospital probable cases, and was the only infected patient who had been in the two wards during the relevant time period. In addition, all the nurses infected had been assigned to care for him during the incubation period of their illness. Strong supportive evidence that could not otherwise be explained by contact with other patients comes from the cluster from radiology department.

## Conclusions

We present this case to highlight the diagnostic as well as public health problems posed by a patient with a rather atypical SARS, whose illness was easily explained by a positive blood culture. Classically, SARS is described as an illness with an incubation of 2 to 7 days followed by a prodrome of high fever with headache, malaise, and myalgia. At the onset of the illness, some patients have mild respiratory symptoms. After 3 to 7 days, a lower respiratory phase with nonproductive cough or dyspnea begins (*8*). Although the clinical signs and symptoms in otherwise healthy persons are widely known, the full clinical spectrum is not known.

In the study by Lee et al., 78% of patients had abnormal chest radiographs at the onset of fever (*4*). Peiris et al. reported that all their patients had radiologic evidence of consolidation at admission (*9*). In another study of 10 cases, 9 had abnormal chest x-rays (*3*). A Canadian study reported that two of nine patients had very subtle chest radiographs. Repeat chest x-rays were read as normal in these two patients (*2*). Without radiographic abnormalities, the diagnosis of SARS can be difficult, especially if a cause for fever exists. By the time the radiographs became abnormal in our patient, he had infected healthcare workers. The implications of such a case and its consequences on the practice of medicine are important, even in current SARS-free areas because of world travel.

Although we are taught to apply Occam’s razor and search for a unifying diagnosis, multiple coexisting conditions are a part of clinical medicine. SARS can coexist with other febrile illnesses. The combination of atypical signs and symptoms and a coexisting diagnosis can have negative public health implications.

Close contact is defined by WHO as having cared for or lived with a SARS patient or having had direct contact with respiratory secretions and body fluids of a SARS patient. This contact history is often difficult to determine and quantify. In one case, the only “contact” elucidated was passing through an emergency department of a hospital with a SARS outbreak (*10*). We are not the first group to have seen atypical SARS in a patient with multiple coexisting conditions (*10*).

In a SARS outbreak, we suggest that all patients with undifferentiated fever or pneumonia be cared for as if they had SARS for the safety of healthcare workers and patients, implying the use of full precautions (N95 respirators, gown, gloves, and goggles) by healthcare workers for all patient-care activities (e.g., ward rounds, baths, wound dressings, performance of radiologic procedures). A powered air purifying respirator should be used when performing aerosol-generating activities, e.g., chest physiotherapy. Patients with undifferentiated fever or pneumonia should be placed in single rooms that meet generally accepted guidelines for the isolation of infected persons (*11*). Establishing an explanation for the fever (e.g., a positive blood culture) in a person with a contact history should not necessitate removing the patient from isolation when a SARS outbreak is ongoing. A detailed contact history should include the travel history of the patient and his family members, as well as of their medical condition, and a much broader definition of contact is necessary, e.g., being in a hospital in which a SARS outbreak occurs. Tests for the SARS-CoV may be ordered, but their low sensitivity must be considered when deciding on the patient’s disposition.

Extreme measures, such as regarding all patients with respiratory infections as potential SARS cases, have also been advocated in other studies (*12*). Nebulizer therapy has been banned in many institutions in Hong Kong, and a protocol for delivering inhaled bronchodilators without nebulization to patients with asthma has been implemented in Singapore General Hospital. Issues, such as bed availability, will need to be weighed against the need to keep patients in isolation rooms. The number of patients that can be cared for will also be lower. The SARS outbreak has focused attention on hygiene standards in our hospitals. Asymptomatic or pauci-symptomatic cases are the norm with most viral infections. With SARS, such patients may still be highly infectious. Infection control measures are needed to prevent similar clusters of infections in the future.
